# Editorial: LRRK2—Fifteen Years From Cloning to the Clinic

**DOI:** 10.3389/fnins.2022.880914

**Published:** 2022-04-11

**Authors:** Hardy Rideout, Elisa Greggio, Arjan Kortholt, R. Jeremy Nichols

**Affiliations:** ^1^Biomedical Research Foundation of the Academy of Athens, Athens, Greece; ^2^Department of Biology, University of Padova, Padua, Italy; ^3^Department of Cell Biochemistry, University of Groningen, Groningen, Netherlands; ^4^YETEM-Innovative Technologies Application and Research Centre, Suleyman Demirel University, Isparta, Turkey; ^5^Centro Studi per la Neurodegenerazione (CESNE), University of Padova, Italy; ^6^Department of Pathology, Stanford University, Stanford, CA, United States

**Keywords:** LRRK2, Parkinson's disease, lysosome, endosome, biomarker, kinase, alpha-synuclein, phosphorylation

In the time since the identification of LRRK2 at the PARK8 locus as the responsible gene mutated in a common autosomal dominantly inherited form of Parkinson's disease (Paisan-Ruiz et al., 2004; Zimprich et al., 2004), it has become increasingly evident that activity of this protein plays a crucial role in disease pathogenesis of Parkinson's disease (PD). Genetic variance within the LRRK2 gene gives rise to PD that generally overlaps clinically and neuropathologically with idiopathic PD (iPD). In the 15+ years since its isolation, we have gone from *in-vitro* assays and hypotheses, to state-of-the-art biomarkers and Phase I trials for treatments.

There are various mechanisms proposed for LRRK2-mutant induced neuropathology in monogenic PD, however it remains unclear if these are the same mechanisms disrupted in iPD. Recent work has yielded two key findings: that LRRK2 activity may also play a major role in multiple forms of PD, not only those associated with mutations in the LRRK2 gene; and secondly, that this activity contributing to the neurodegeneration underlying PD occurs in both neuronal cells, as well as non-neuronal cells (e.g., microglia/macrophages, astrocytes, peripheral immune cells).

This Research Topic provides a comprehensive collection of articles illustrating the role of LRRK2 in the physiology of neuronal and non-neuronal cells as well as the impacts of mutant LRRK2 in PD pathology, ranging from mechanisms, biomarkers and therapeutic opportunities. The articles provide a review of the literature as well as novel data around LRRK2 biochemical properties and cellular mechanisms in the context of endolysosomal system, synaptic function and immune-related pathways.

## In Which Cells Does LRRK2 Exert Its Normal and Mutant Pathogenic Effects? The Role of LRRK2 in Neurons vs. Immune Cells

It is increasingly clear that PD is a multisystem disorder not only affecting dopaminergic neurons but also other neuronal types as well as non-neuronal cells, both in the brain and in peripheral tissues (Langston et al., 2015). Central and peripheral inflammation may act as contributing factors for disease onset or progression and accumulating evidence points to the gut-brain axis as an important route in disease. LRRK2 acts as a positive regulator of inflammatory pathways and, while a beneficial or detrimental outcome of LRRK2 mutations may depend on the specific inflammatory condition, one current hypothesis is that LRRK2 PD mutations exacerbate the brain inflammatory state, accelerating the neurodegenerative process. In this Research Topic, Rastegar and Dzamko provide a thorough overview of the physiological and pathological functions of LRRK2 in innate immunity with particular focus on Toll-like receptor signaling and inflammasome. Along these lines, Cabezudo et al. discuss how mutant LRRK2-driven inflammation may trigger PD starting from peripheral organs such as the gut and immune circulating cells, according to a multiple-hit hypothesis for PD. The role that LRRK2 plays in immunity and inflammation is also examined by Wallings et al., providing an insightful overview of LRRK2 function in peripheral organs and how systemic inflammation could trigger or accelerate PD. Finally, Aasly reports that in a cohort of 100 Norwegian LRRK2 G2019S carriers, the presence of G2019S increases the incidence of inflammatory diseases such as rheumatoid arthritis and multiple sclerosis, further supporting the connection between PD and inflammation.

## Where in the Cell Does LRRK2 Exert Its Normal and Mutant Pathogenic Effects? The Function of LRRK2 at the Synapse and Endolysosomal System

Lysosomal dysfunction and the resultant impaired clearance and recycling of proteins is a core causal hypothesis for many proteinopathies, including the synucleinopathy, PD. A compromised endolysosomal system is implicated in PD by multiple disease linked genomic loci encoding proteins involved in vesicular trafficking, endocytosis, lysosomal function, and mitophagy (Bandres-Ciga et al., 2020; Erb and Moore, 2020). Biochemical and cell biological studies in a variety of cell types (neuronal and non-neuronal) place LRRK2 in the regulation of endolysosomal trafficking and autophagy. One mechanism for this to occur is via LRRK2 phosphorylation of Rab GTPases, which when altered by mutant-LRRK2 activity, results in vesicle/organelle defects observed in these various experimental contexts. Kuwahara and Iwatsubo discuss how LRRK2 signaling to multiple Rab GTPases implicate LRRK2 kinase dysfunction as a driver of endolysosomal pathomechanisms in PD. Focusing on the role of LRRK2 in autophagy, Madureira et al. carefully describe the normal and disease-associated role of LRRK2 on autophagic function, from direct regulation of phagophore formation to autolysosome fusion. In an experimental demonstration of the intersection between LRRK2, the endolysosome system and genetics, Sanyal et al. demonstrate LRRK2 inhibition can restore some of the impaired lysosome function observed in GBA heterozygous iPSC neurons.

## LRRK2 Animal Models and It's Role at the Synapse

There is a diverse array of rodent models that have been developed to study the biology of LRRK2, ranging from knockouts, knockin mutations, transgenic overexpression, viral vector delivery and inoculation of pre-formed alpha-synuclein fibrils. These models have been crucial at enabling identification of neuronal and peripheral LRRK2 related phenotypes. Seegobin et al. provides a careful review of the LRRK2 rodent models and the phenotypes they display at locomotion and behavior, dopamine system, and electrophysiology and then discuss similar phenotypic themes in *Drosophila, Caenorhabditis elegans, and Danio rerio*, all with a single *LRRK* homolog. Dues and Moore provide a detailed review of the rodent model evidence for the role of LRRK2 in protein aggregation as it relates to human disease.

Although no LRRK2 animal model recapitulates all of the cardinal features of PD, the neurophysiological changes, dopamine dysregulation and modest behavioral changes indicate a compromised synaptic environment. In post-mitotic neurons, dysregulated movement of vesicles would not only impact protein recycling in the cell body, but also could induce synaptic phenotypes by disruption of transport to and away from the synapse and mis-trafficking of ion-channels and neurotransmitter receptors or the uptake of their substrates. Kuhlmann and Milnerwood discuss the current state of understanding of the role of LRRK2 at the synapse, providing insights from electrophysiological phenotypes in pre-clinical LRRK2 mutant rodent models. LRRK2 mutations are carried throughout life, but their pathological consequences are observed only late in life. Huntley and Benson describe how the early disruptions in synaptic activity and plasticity, present throughout development, impairs the establishment or maturity of brain circuitry.

## What Is the Structure of LRRK2 and How Is It Regulated by PTM, Binding Partners and Other Mechanisms?

Structural studies on LRRK2 have been ongoing for almost two decades now. A major breakthrough in the field of structural biology in general and for determining the LRRK2 structure has been the development in electron microscopy (EM). In this issue, Taylor et al. discuss the implications of the recently identified Roc-COR-Kinase-WD40 (RCKW) structures for the complex LRRK2 activation mechanism (Deniston et al., 2020; Watanabe et al., 2020). Among others they discuss the cross-talk between the different LRRK2 domains and propose a mechanism by which the kinase domain, along with key phosphorylation sites, can serve as an allosteric hub for mediating conformational changes. Indeed, a more recent high resolution cryo-EM structure of full-length LRRK2 confirms that both the N-terminus and C-terminus are in direct contact with the kinase domain and most likely play an important role in regulating the kinase activity (Myasnikov et al., 2021). Several phosphorylation and protein-protein interactions (PPIs) within these domains regulate the conformation and activity of LRRK2. As discussed in detail by Marchand et al. in this issue, several studies indicate that LRRK2's phosphorylation is regulating LRRK2 activity and localization and therefore plays an important role in its pathological and physiological functioning. Phosphorylation of LRRK2 S910/S935 within the N-terminus of LRRK2 has been shown to be important for binding to 14-3-3 and regulating the cellular localization of LRRK2. Manschwetus et al. provide in this issue a quantitative analysis of the interaction between all human 14-3-3 isoforms and LRRK2, including both known and the discovery of new 14-3-3 binding sites. In addition to 14-3-3, over the years numerous PPIs have been identified for LRRK2, which are in this issue summarized by Gloeckner and Porras. In addition, they have analyzed the previously published LRRK2 interactome maps and discussed these in the perspective of putative LRRK2 functions. O'Hara et al. discuss in detail the nature of the interaction between LRRK2 and α-synuclein. An initial paper has suggested that mutant G2019S LRRK2 can directly interact with and phosphorylate α-synuclein. Indeed, substantial experimental evidence points toward an interplay between LRRK2 and α-synuclein, however O'Hara et al., conclude that the interactions between LRRK2 and α-synuclein are likely to be indirect, most likely with Rab proteins and chaperones as mediators.

## LRRK2 Based Therapeutics and Biomarkers

This latter issue has attained greater urgency since early Phase I clinical trials of LRRK2 kinase inhibitors, potential therapeutic candidates in PD, have begun (clinicaltrials.gov/ct2/show/study/NCT03710707; clinicaltrials.gov/ct2/show/NCT03976349). New precision medicine approaches targeting the most prevalent mutant, LRRK2-G2019S, have been established as well (Garofalo et al., 2020; Lesniak et al., 2021). Parallel to these efforts, trials of investigational compounds targeting LRRK2 activity must also be accompanied by validated LRRK2 biomarkers. Here, the goal is two-fold: to establish a marker of changes in LRRK2 function that correlate with, or predict, disease progression; and, assays capable of demonstrating target engagement of test compounds. Several contributions to this Research Topic address these specific areas.

The issue of sensitive and standardized LRRK2, and LRRK2 pathway, detection and quantification in clinical biofluids remains a key unmet need in the PD field. In this Research Topic, three individual submissions address this critical area from distinct perspectives. In a more broadly focused review from Rideout et al., the principal uses of LRRK2-focussed biomarkers (e.g., pharmacodynamic outcome measures, disease severity/patient stratification, and progression) are introduced, highlighting the current assays being developed and implemented. In a contribution from the group of Mabrouk et al., a novel methodology is described employing a Stable Isotope Standard Capture by Antipeptide Antibody (SISCAPA) based assay to measure and quantify endogenous wild type and mutant (G2019S) LRRK2 in clinically relevant biofluids such as CSF. The approach represents a great advance in the quantitative determination of changes in LRRK2 levels, and can be further developed to include additional targets including substrates of LRRK2 and auto-phosphorylated LRRK2 residues. Finally, from the group of Kelly and West comes a report discussing the key role biomarkers fill in clinical trials, particularly in the case of LRRK2-PD where outcome measures of LRRK2 kinase activity are key indicators of response to therapies targeting this function [e.g., small molecule inhibitors or antisense oligonucleotides (ASOs)].

While significant progress has been made in the LRRK2 biomarker space, especially at the single-plexed level, one potential direction for the field would be the adoption of true multiplexed assays, quantifying multiple targets in parallel, to establish a more thorough “picture” of LRRK2, and LRRK2 pathway, status. In order for such assays to be widely deployed, large longitudinal studies (of both familial and idiopathic PD) are critically needed, defining patterns of activation at each stage of the disease, and in multiple biofluid sample types. The implementation of sensitive standardized quantitative assays that can be adapted to the multiple uses introduced and discussed in this Research Topic should be a primary goal of the field moving forward.

## Conclusion

In 15 years, the field has made great strides in assaying, detecting, targeting and understanding LRRK2. This Research Topic presents the cell type, where in the cell, and how LRRK2 itself is structured and regulated during both its physiological function and in PD. These articles have revealed themes of phenotypes amongst LRRK2 model systems implicating LRRK2 in a variety of cellular processes, undoubtably there are many more yet to be elucidated. With the introduction of LRRK2 targeting drugs in clinical trials, current state of understanding the normal and mutant role of LRRK2 is paramount.

## Author Contributions

All authors contributed to the editorial tasks of the Research Topic and wrote the manuscript. All authors contributed to the article and approved the submitted version.

## Funding

RN was supported by the Alexander and Eva Nemeth Foundation, The Farmer Family Foundation Parkinson's Research Initiative, The Sergey Brin Family Foundation and The Michael J. Fox Foundation. EG was funded by the Michael J. Fox Foundation and intramural funding from the University of Padova. AK was funded by The Michael J. Fox Foundation, Stichting ParkinsonFonds and a TUBITAK 2232 fellowship.

## Conflict of Interest

The authors declare that the research was conducted in the absence of any commercial or financial relationships that could be construed as a potential conflict of interest.

## Publisher's Note

All claims expressed in this article are solely those of the authors and do not necessarily represent those of their affiliated organizations, or those of the publisher, the editors and the reviewers. Any product that may be evaluated in this article, or claim that may be made by its manufacturer, is not guaranteed or endorsed by the publisher.

## References

[B1] Bandres-Ciga S. Saez-Atienzar S. Kim J. J. Makarious M. B. Faghri F. Diez-Fairen M.. (2020). Large-scale pathway specific polygenic risk and transcriptomic community network analysis identifies novel functional pathways in Parkinson disease. Acta Neuropathol. 140, 341–358. 10.1007/s00401-020-02181-3 32601912 PMC8096770

[B2] Deniston C. K. Salogiannis J. Mathea S. Snead D. M. Lahiri I. Matyszewski M.. (2020). Structure of LRRK2 in Parkinson's disease and model for microtubule interaction. Nature 588, 344–349. 10.1038/s41586-020-2673-2 32814344 PMC7726071

[B3] Erb M. L. Moore D. J. (2020). LRRK2 and the endolysosomal system in Parkinson's disease. J. Parkinsons Dis. 10, 1271–1291. 10.3233/JPD-202138 33044192 PMC7677880

[B4] Garofalo A. W. Bright J. De Lombaert S. Toda A. M. A. Zobel K. Andreotti D.. (2020). Selective inhibitors of G2019S-LRRK2 kinase activity. J. Med. Chem. 63, 14821–14839. 10.1021/acs.jmedchem.0c01243 33197196

[B5] Langston J. W. Schule B. Rees L. Nichols R. J. Barlow C. (2015). Multisystem Lewy body disease and the other parkinsonian disorders. Nat. Genet. 47, 1378–1384. 10.1038/ng.3454 26620112

[B6] Lesniak R. K. Nichols R. J. Schonemann M. Zhao J. Gajera C. R. Fitch W. L.. (2021). Discovery of G2019S-selective leucine rich repeat protein kinase 2 inhibitors with in vivo efficacy. Eur. J. Med. Chem. 229, 114080. 10.1016/j.ejmech.2021.114080 34992038

[B7] Myasnikov A. Zhu H. Hixson P. Xie B. Yu K. Pitre A.. (2021). Structural analysis of the full-length human LRRK2. Cell 184, 3519–27.e10. 10.1016/j.cell.2021.05.004 34107286 PMC8887629

[B8] Paisan-Ruiz C. Jain S. Evans E. W. Gilks W. P. Simon J. van der Brug M.. (2004). Cloning of the gene containing mutations that cause PARK8-linked Parkinson's disease. Neuron 44, 595–600. 10.1016/j.neuron.2004.10.023 15541308

[B9] Watanabe R. Buschauer R. Bohning J. Audagnotto M. Lasker K. Lu T. W.. (2020). The in situ structure of Parkinson's disease-linked LRRK2. Cell 182, 1508–18.e16. 10.1016/j.cell.2020.08.004 32783917 PMC7869717

[B10] Zimprich A. Biskup S. Leitner P. Lichtner P. Farrer M. Lincoln S.. (2004). Mutations in LRRK2 cause autosomal-dominant parkinsonism with pleomorphic pathology. Neuron 44, 601–607. 10.1016/j.neuron.2004.11.005 15541309

